# Fishbone-Induced Appendicular Perforation: A Rare Case Report of Amyand’s Hernia

**DOI:** 10.7759/cureus.37313

**Published:** 2023-04-08

**Authors:** Anirban Das, Vikas Pandurangappa, Sushant Tanwar, Sajith K Mohan, Harish Naik

**Affiliations:** 1 Surgery, Vardhman Mahavir Medical College and Safdarjung Hospital, New Delhi, IND

**Keywords:** small-bowel obstruction, impacted fishbone in appendix, exploratory laporotomy, strangulated inguinal hernia, appendicular perforation, amyand’s hernia

## Abstract

Amyand’s hernia is a rare type of hernia where the appendix is found to be the content of the inguinal hernial sac. It is most often diagnosed intraoperatively wherein the appendix may be found healthy, incarcerated, inflamed, or perforated. Claudius Amyand performed a successful appendectomy on a patient with an appendix noted in the inguinal canal and this condition was hence named after him. The incidence of Amyand's hernia is rare in inguinal hernia patients. There are no defined guidelines for the management of Amyand's hernia but adequate resuscitation followed by immediate appendectomy is widely followed. Here is a case report of a 60-year-old male presenting to the Emergency Department with an irreducible right-side inguinal hernia with features of small bowel obstruction. On exploration, Amyand's hernia was identified with appendicular tip perforation due to an impacted fishbone with pyoperitoneum. Appendectomy was done through midline laparotomy with impacted fishbone removal from the hernial sac with tissue repair of the hernia. There are as such no reported cases of fishbone-induced appendicular perforation in an Amyand's hernia in the available literature. After the exploration, we found the management of the case challenging regarding the closure of the hernia.

## Introduction

A hernia is defined as the protrusion of an organ or its fascia through an abnormal opening in the wall containing it [1]. Amyand’s hernia is a rare type of hernia when the appendix is found to be the contents of the inguinal hernial sac. Only about 0.19-1.7% of all inguinal hernia cases turn out to be Amyand’s hernia [2-5]. Diagnosing an Amyand’s hernia is difficult preoperatively and is most often seen intraoperatively wherein the appendix may be found healthy, incarcerated, inflamed or perforated [6]. This condition of an appendix noted in an inguinal hernial sac was named after Claudius Amyand, who performed a successful appendectomy on a boy who presented with a right inguinal hernia [7]. He had found a pin within the appendix, encrusted with a stone. A classification system had been proposed by Losanoff and Basson for the guidance of treatment in Amyand’s hernia [8]. It is classified into four types: Type 1 being normal appendix and Types 2-4 being acute appendicitis with other complications. We are reporting a rare case of fishbone-induced appendicular perforation in an Amyand’s hernia which is the first of its kind.

## Case presentation

A 60-year-old male rickshaw puller presented to the surgical emergency with right inguinoscrotal swelling and acute onset of pain and fever for three days, and features of intestinal obstruction for one day. He had a history of right inguinoscrotal swelling for the last two years which was reducible in a lying down position but became irreducible for the last six months. He had no medical comorbidities or surgical intervention in the past but habituates to tobacco smoking and alcohol intake for the past 30 years.

**Figure 1:**
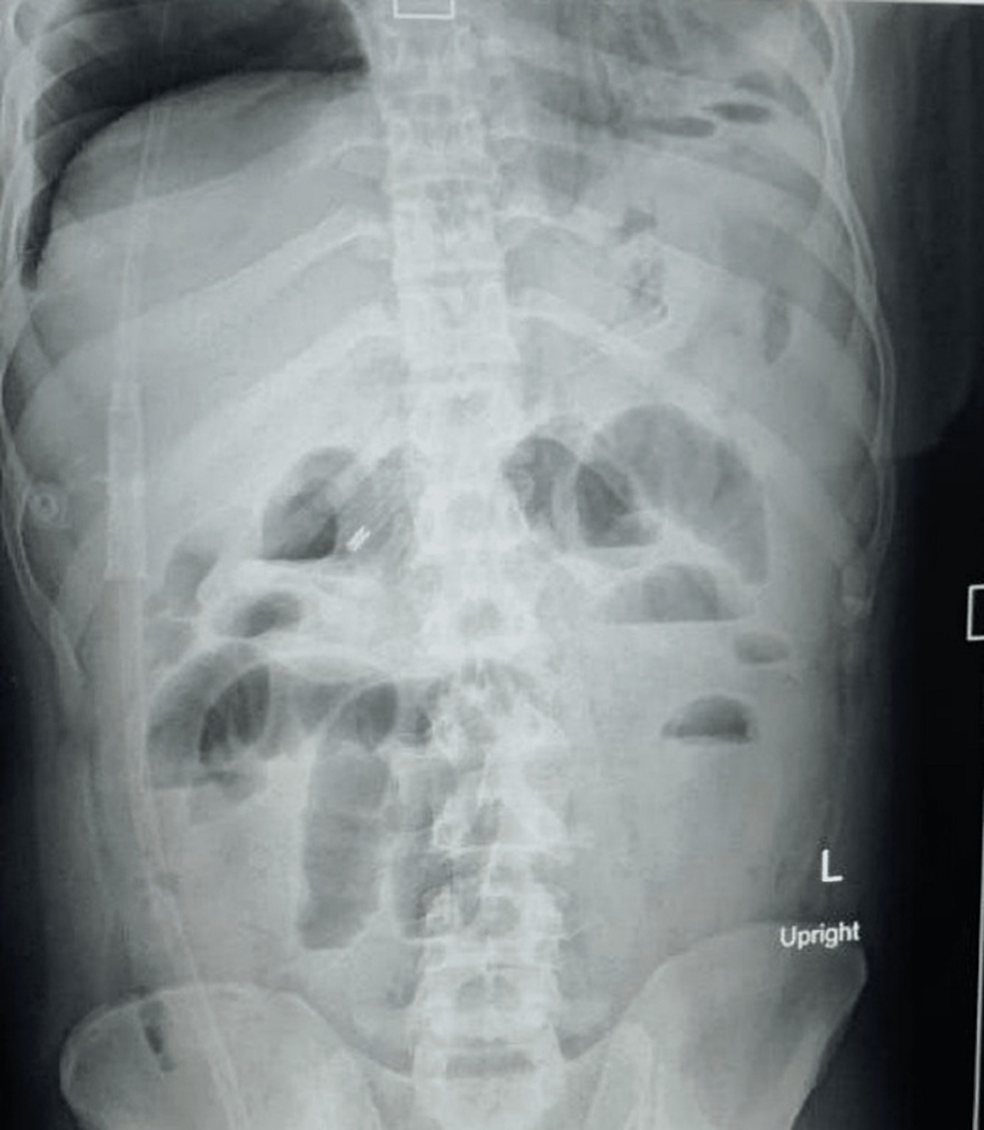
Multiple air fluid levels suggestive of acute intestinal obstruction.

On clinical examination, the patient was afebrile and dehydrated, with tachycardia and hypotension. Abdominal examination revealed a distended abdomen with no tenderness, guarding, or rebound tenderness; bowel sounds were exaggerated. There was a 6x6 cm irreducible inguinoscrotal swelling reaching the bottom of the right hemiscrotum. The swelling was tender and the overlying skin was erythematous with separately palpable right testis. The left hernial site, testis, and digital rectal examination were normal. Abdominal x-ray showed multiple air-fluid levels suggesting small bowel obstruction (Figure 1).

Ultrasonography (USG) whole abdomen showed free echogenic fluid in the pelvis with multiple dilated small bowel loops more than 3.8 centimeters in diameter. USG inguinoscrotal region revealed a right incarcerated indirect hernia sac with a non-peristaltic, blind-ending tubular structure with fluid collection noted as content. The vascularity of both testes was normal. Hematological examination showed a total leucocyte count of 25,000/µl (normal: 4,000-11,000/$\mu$l), serum creatinine of 2 mg/dl (normal: 0.7-1.3 mg/dl), with other routine investigations within normal limits, and viral serology was non-reactive.

He was diagnosed as a case of acute intestinal obstruction due to right incarcerated indirect inguinal hernia with acute kidney injury. After initial fluid resuscitation, he was posted for emergency right inguinoscrotal exploration under general anesthesia with written informed consent.

**Figure 2:**
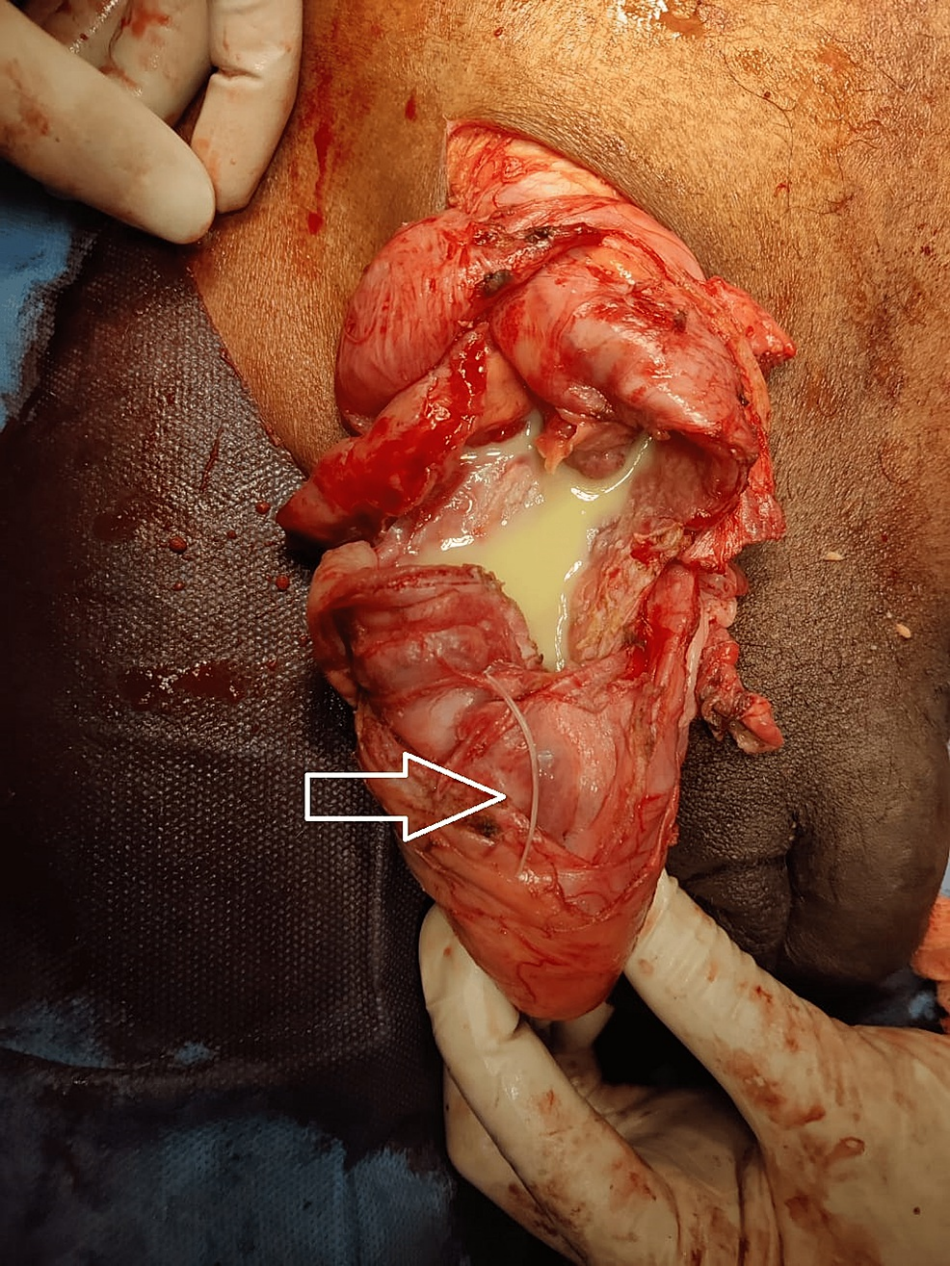
Pool of pus noted in hernial sac with impacted fishbone (white arrow).

**Figure 3:**
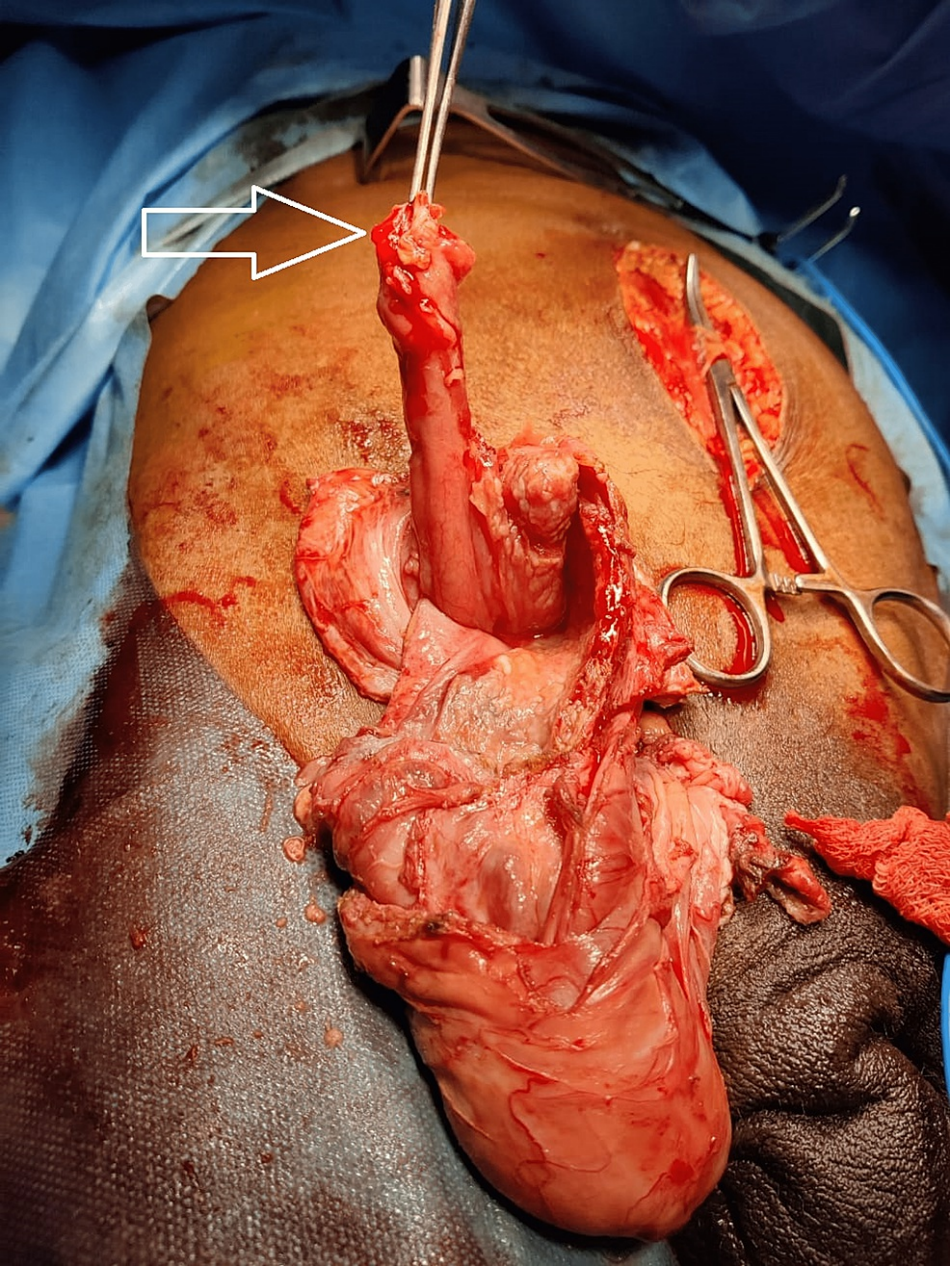
Perforated appendix (white arrow) found within hernial sac.

On exploration, there was a chronic right inguinal hernia sac with thick walls and dense adhesions, separate from the spermatic cord, and testis. On opening the hernia sac, nearly 300 ml of pus was drained and an impacted fishbone of 4 cm was removed from inside the sac (Figure 2). A 10 cm long inflamed edematous appendix was noted as the content of the hernia with phlegmon formation (Figure 3).

**Figure 4:**
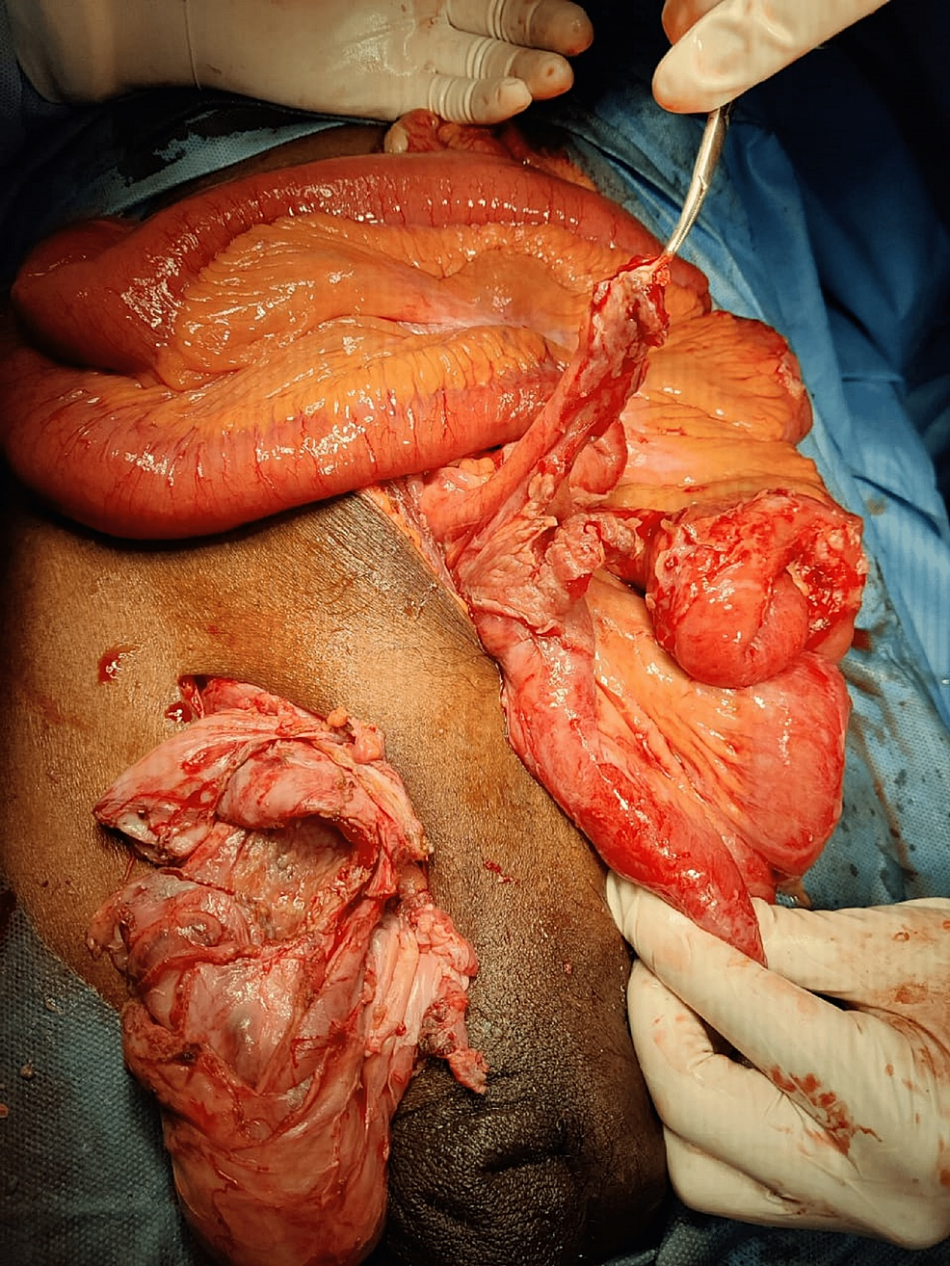
Midline laparotomy done and appendix delivered through midline incision.

An infra umbilical midline exploratory laparotomy was done. Upon exploration, there was continuous drainage of pus from the pelvis. The appendix and the phlegmon were delivered into the midline incision (Figure 4).

Phlegmon was dissected meticulously to separate the appendix from the omentum. The tip of the appendix was perforated. The base of the appendix, the caecum, and the rest of the viscera was healthy. Appendectomy was done and the specimen was sent for histopathological examination. Peritoneal lavage was given with warm normal saline. The hernia sac was transfixed at the deep ring, excised, and sent for biopsy.

Hemostasis was secured, the left pelvic drain was placed and the midline was closed in layers. Over the hernia site, Bassini's tissue repair was done. The inguinoscrotal incision was closed in layers placing a negative suction inside the right hemi-scrotum. The patient was extubated and was placed on antibiotic and analgesic support. Enhanced Recovery After Surgery (ERAS) protocol followed. On the second postoperative day, he was allowed per mouth and the pelvis and scrotal drains were removed. He developed a superficial surgical site infection over the inguinoscrotal incision (Southampton grade 2) which was managed by a regular sterile dressing. He was discharged upon normalization of serum creatinine on the fourth postoperative day. Skin sutures were removed after 10 days. The patient is on a regular follow-up which has been uneventful.

## Discussion

Appendix in a hernial sac is very unusual and was first reported in a femoral hernia in 1731 by De Garengeot [9]. Claudius Amyand, an English sergeant surgeon, diagnosed a non-reducible inguinal hernia with an appendix as its content on an 11-year-old boy and performed the first appendectomy in 1753 [7]. The incidence of Amyand’s hernia is 0.19-1.7% of all the reported cases of Inguinal hernia [2-5]. It is three times more common in children, due to the patency of the processus vaginalis and in males and is mostly observed on the right side [10]. The clinical signs of acute appendicitis are usually absent in these cases. The diagnosis of an appendix in a hernial sac is usually made intraoperatively. The appendix can also rarely be found in other types of hernias (Table 1) [11].

**Table 1:** Appendix found in different types of hernia.

Type of hernia	No.
Incarcerated inguinal hernia	113
Strangulated inguinal hernia	11
Incarcerated femoral hernia	30
Strangulated femoral hernia	11
Umbilical hernia	4
Strangulated obturator hernia	3
Incarcerated spiegelian hernia	3
Strangulated diaphragmatic hernia	7
Incisional hernia	2
Epigastric hernia	2
Strangulated internal hernia	1
Strangulated paraduodenal hernia	1
Total	188

Different theories have been proposed for the occurrence of Amyand’s hernia. External compression of the appendix at the hernial neck can be the cause of inflammation [9]. Due to a long appendix pointing towards the groin or loose peritoneal reflections and redundant cecum, the appendix may reach the hernia and get stuck in the sac [12].

Sudden onset periumbilical pain with a tender irreducible mass in the inguinoscrotal region is usually the common presentation of an Amyand’s hernia [13]. It can result in various complications such as a perforated appendix with peri appendicular or intra-abdominal abscess, necrotizing fasciitis of the anterior abdominal wall, epididymal-orchitis or testicular abscess, and rarely an in situ testicular arterial thrombosis. Mortality has been reported in the range of 6-15% [14,15]. In our case the cause of the appendicular perforation could either be due to strangulation or due to impaction of the fishbone and resulting infection, but it isn’t easy to prove the exact mechanism for perforation. Probably, the fishbone had come out of the appendicular lumen through the perforation and was present in the pus-filled hernia sac.

Management of Amyand’s hernia is very important. We can follow the widely accepted classification of Amyand's hernia which is Nikki’s modification of Losanoff and Basson (Table 2) [8].

**Table 2:** Nikki's modification of Losanoff and Basson classification. [8]

Classification	Description	Surgical management
Type 1	Normal appendix within inguinal hernia	Hernia reduction, mesh repair, appendectomy through separate incision
Type2	Acute appendicitis within inguinal hernia without abdominal sepsis	Appendectomy through hernia, primary tissue repair without mesh
Type3	Acute appendicitis within inguinal hernia with abdominal wall or peritoneal sepsis	Appendectomy through laparotomy, primary tissue repair without mesh
Type 4	Acute appendicitis within inguinal hernia with related or unrelated abdominal pathology	Manage as 1-3, evaluate other pathology
Type 5a	Normal appendix within incisional hernia	Appendectomy through incisional hernia and mesh repair
Type 5b	Acute appendicitis within incisional hernia without abdominal or peritoneal sepsis	Appendectomy through incisional hernia and primary tissue repair
Type 5c	Acute appendicitis within incisional hernia with abdominal or peritoneal sepsis	Manage as type 4

A lower midline approach is followed for cases with suspicion of perforation or pelvic abscess with hernial repair usually completed during the procedure or delayed because of inflammation [13]. Primary tissue repair is usually advocated for hernial repair in cases of an inflamed or perforated appendix. However, there are case reports showing mesh repair with no significant increase in infection rates and post-operative recurrences [13,16].

## Conclusions

Amyand’s hernia is a rare entity with a varying presentation, usually presenting as a non-reducible hernia with or without small bowel obstruction. The preoperative clinical diagnosis in such cases is difficult. A surgeon must be prepared to encounter such unusual pathology while doing hernia surgery. Management of Amyand’s hernia depends on the skill of the operating surgeon, the clinical scenario of the patient, and the inflammatory status of the appendix.
